# Zika Virus Non-Structural Protein NS5 Inhibits the RIG-I Pathway and Interferon Lambda 1 Promoter Activation by Targeting IKK Epsilon

**DOI:** 10.3390/v11111024

**Published:** 2019-11-04

**Authors:** Rickard Lundberg, Krister Melén, Veera Westenius, Miao Jiang, Pamela Österlund, Hira Khan, Olli Vapalahti, Ilkka Julkunen, Laura Kakkola

**Affiliations:** 1 Institute of Biomedicine, University of Turku, Kiinamyllynkatu 10, 20520 Turku, Finland; rickard.lundberg@utu.fi (R.L.); hira.khan@utu.fi (H.K.); ilkka.julkunen@utu.fi (I.J.); 2 Expert Microbiology Unit, National Institute for Health and Welfare, Mannerheimintie 166, 00300 Helsinki, Finland; krister.melen@thl.fi (K.M.); veera.westenius@thl.fi (V.W.); miao.jiang@helsinki.fi (M.J.); pamela.osterlund@thl.fi (P.Ö.); 3 Department of Virology, University of Helsinki, Haartmaninkatu 3, 00290 Helsinki, Finland; olli.vapalahti@helsinki.fi; 4 Clinical Microbiology, Turku University Central Hospital, Kiinamyllynkatu 10, 20520 Turku, Finland

**Keywords:** Zika virus, innate immunity, interferon, IKK epsilon

## Abstract

The Zika virus (ZIKV) is a member of the *Flaviviridae* family and an important human pathogen. Most pathogenic viruses encode proteins that interfere with the activation of host innate immune responses. Like other flaviviruses, ZIKV interferes with the expression of interferon (IFN) genes and inhibits IFN-induced antiviral responses. ZIKV infects through epithelial barriers where IFN-λ1 is an important antiviral molecule. In this study, we analyzed the effects of ZIKV proteins on the activation of IFN-λ1 promoter. All ZIKV proteins were cloned and transiently expressed. ZIKV NS5, but no other ZIKV protein, was able to interfere with the RIG-I signaling pathway. This inhibition took place upstream of interferon regulatory factor 3 (IRF3) resulting in reduced phosphorylation of IRF3 and reduced activation of IFN-λ1 promoter. Furthermore, we showed that ZIKV NS5 interacts with the protein kinase IKKε, which is likely critical to the observed inhibition of phosphorylation of IRF3.

## 1. Introduction

The Zika virus (ZIKV) is a flavivirus that was discovered in 1947 near Entebbe, Uganda. In humans, ZIKV infection has been associated with a mild disease characterized by fever, rash, arthritis, and conjunctivitis [1]. However, the recent epidemic in the Americas in 2013–2015 in an immunologically naïve population revealed that ZIKV can also cause severe neurological symptoms, such as Guillain–Barré syndrome and microcephaly [2]. ZIKV is primarily spread by *Aedes* species mosquitoes, and vertical and sexual transmission among humans was confirmed during the last epidemic. Multiple infection routes of ZIKV are facilitated by its ability to productively infect several types of human cells, such as skin fibroblast and dendritic cells [3], Sertoli cells [4], trophoblast progenitor cells and cytotrophoblasts, as well as placental macrophages [5,6]. ZIKV also replicates in human brains and cells of the neuronal origin [7,8].

Infected cells respond to virus infections by activating innate immune responses. In RNA virus infection, especially the RIG-I-like pattern recognition receptors, RIG-I and MDA5 recognize ssRNA and dsRNA molecules of invading and replicating viruses. RIG-I-like receptors activate signaling cascades involving cellular kinases that eventually phosphorylate and activate transcription factors IRF3, IRF7, and NF-ĸB. These factors translocate into the nucleus and initiate the expression of type I (IFN-α/β) and type III interferon (IFN-λ1-4) and other inflammatory cytokine genes. IFN-α/β is produced by several cell types, whereas IFN-λs are produced by immune cells and cells of epithelial origin [9]. Virus-infected cells secrete IFNs that bind to their specific receptors, IFNAR1–IFNAR2 (IFN-α/β) and IL28Ra–IL10Rb (IFN-λ1-4) initiating a signaling cascade leading to the phosphorylation, activation, and dimerization of transcription factors STAT1 and STAT2. STAT1/STAT2 dimers associate with IRF9 to form the so-called ISGF3 complex, which then translocates into the nucleus where it activates the transcription of interferon-stimulated genes (ISGs). This initiates cellular antiviral responses via the production of antiviral proteins such as MxA, Viperin, and IFIT proteins [10,11,12,13]. Most, if not all, pathogenic viruses encode proteins that interfere with the activation host innate immune responses.

ZIKV has a positive sense ssRNA genome that encodes for one large polyprotein including three structural (C, M, and E) proteins and seven non-structural (NS1, NS2A, NS2B, NS3, NS4A, NS4B, and NS5) proteins. The polyprotein is cleaved by cellular and viral proteases into individual proteins. ZIKV infection leads to the production of interferons and antiviral proteins [14] and ZIKV RNA has been shown to stimulate both RIG-I and MDA5 receptors [15]. In order to replicate more efficiently in virus-infected cells, ZIKV has mechanisms to evade or delay the activation of innate immune responses. Recent studies have shown that ZIKV can interfere with interferon-induced responses: ZIKV infection inhibits STAT1 and STAT2 phosphorylation [16], and especially ZIKV NS5 protein inhibits STAT1 phosphorylation [15] and induces the proteasomal degradation of STAT2 [15,17,18,19]. ZIKV NS2B-NS3 protein complex promotes the degradation of Jak1 resulting in reduced STAT1 phosphorylation [20]. ZIKV E, NS4A, and NS5 proteins inhibit the expression of IFIT1 gene [18], and NS1, NS2B3, NS4B, and NS5 proteins have been shown to inhibit the expression of IFIT2 gene [20]. In addition, NS1, NS2B, NS4A, and NS5 proteins inhibit IFN-β-induced, and NS5 protein also IFN-λ1-induced ISRE activation [15,18,19]. ZIKV also interferes with the production of IFNs: ZIKV infection prevents the translation of type I and III IFNs in dendritic cells [16]. Several ZIKV proteins (NS1, NS2A, NS2B, NS4A, NS4B, NS5) have also been shown to inhibit the activation of IFN-β promoter [15,18,20,21,22]. NS1, NS4A, and NS5 proteins were demonstrated to inhibit the activation of IRF3 and NS5 was shown to inhibit NF-κB reporters [18]. In most of the events described above the exact molecular mechanisms are not known. However, it has been suggested that some ZIKV proteins block TBK1 function [20,21,22] leading to reduced IRF3 phosphorylation [21].

In the present study, we analyzed the potential inhibitory effect of individual ZIKV proteins on the activation of interferon promoters, specifically, a less-well studied type III IFN-λ1 promoter. We found that the ZIKV NS5 protein efficiently inhibits RIG-I-induced IRF3 phosphorylation, leading to a reduction in type I and type III interferon promoter activation. We show here that ZIKV NS5 interacts with IKKε, an important downstream kinase of the RIG-I pathway. The data indicates that this interaction leads to impaired ability of IKKε to phosphorylate and activate IRF3 leading to reduced activation of IFN promoters including that of the IFN-λ1 gene.

## 2. Materials and Methods

### 2.1. Cloning of ZIKV Genes and Construction of Expression Plasmids

Viral genes coding for structural proteins (capsid, pro-membrane, membrane, and envelope) were constructed synthetically (GeneArt, Thermo Fisher Scientific, Waltham, MA, USA) as one continuous sequence, based on Zika virus isolate BeH819966 (GenBank accession no: KU365779.1). The individual structural genes were subsequently amplified by PCR for expression constructs using the Phusion high-fidelity DNA polymerase (Thermo Fisher Scientific, Waltham, MA, USA) with time and temperature parameters adjusted according to the manufacturer’s instructions. The non-structural genes were either constructed synthetically (NS1 and NS5) or amplified from complementary DNA generated by RT-PCR from viral RNA that was isolated from a Zika virus stock propagated for four days in Vero E6 cells with inoculum of 10^-3^ of isolate FB-GWUH-2016 (Genbank accession no: KU870645.1). The non-structural genes were amplified with the Phusion high-fidelity DNA polymerase (Thermo Fisher Scientific, Waltham, MA, USA) or DreamTaq DNA polymerase (Thermo Fisher Scientific, Waltham, MA, USA). The NS5 MTase and RdRp subunits were amplified with DreamTaq DNA polymerase (Thermo Fisher Scientific, Waltham, MA, USA) and Phusion high-fidelity DNA polymerase (Thermo Fisher Scientific, Waltham, MA, USA), respectively. Primers used in this study are listed in Supplementary Materials Table S1. ZIKV genes were subcloned into a mammalian expression plasmid pEBB-HA (kindly provided by Drs. D. Baltimore and K. Saksela) which contains an N-terminal HA-tag.

Luciferase reporter plasmids pIFN-β-Luc, pIFN-λ1-Luc, pIFN-λ1-ISREmut-Luc, pIFN-λ1-NF-κBmut-Luc, and pMxA-Luc with the indicated promoter areas for the cytokine genes upstream of firefly luciferase gene, Renilla luciferase gene under Rous sarcoma virus promoter (RSV-Renilla), and expression plasmids for the wild type (wt) RIG-I, wtIRF3, the constitutively active form of RIG-I (∆RIG-I), FLAG-tagged MAVS, FLAG-tagged TBK-1, FLAG-tagged IKKε, constitutively active form of IRF3 (IRF3-5D), and FLAG-tagged HCV NS3/4A have been described previously [23,24,25,26,27,28]. All plasmids were prepared for transfections with endotoxin-free maxipreps (Sigma-Aldrich, St. Louis, MO, USA or Qiagen, Hilden, Germany).

For the baculoviral expression of GST-tagged ZIKV NS5, the pBVboost vector [29] was modified to create a GST-pBVboost vector by replacing the *Bam*HI–*Hind*III multiple cloning site with a segment from the pGEX-2T(+) bacterial expression vector (GE Healthcare Europe GmbH, Finland; GenBank: U13850.1). Inserted segment encodes the glutathione S-transferase gene and contains a thrombin recognition site and the multiple cloning site with a *Bam*HI recognition site. The production of pAc/YM1-based GST, GST-NS2 (HCV) and GST-NP (IAV) baculoviral expression plasmids have been described previously [30].

### 2.2. Antibodies

Rabbit anti-IRF3 (FL-425; Santa Cruz Biotechnology, Dallas, TX, USA), mouse anti-IRF3 (SL-12; Santa Cruz Biotechnology, Dallas, TX, USA), rabbit anti-phospho-IRF3 (Ser396) (4D4G; Cell Signaling Technology, Danvers, MA, USA), mouse anti-HA1.1 Epitope Tag (BioLegend, San Diego, CA, USA), mouse anti-GAPDH (6C5; Santa Cruz Biotechnology, Dallas, TX, USA), mouse anti-FLAG (M2; Sigma-Aldrich, St. Louis, MO, USA), rabbit anti-TBK-1/NAK (Cell Signaling Technology, Danvers, MA, USA), rabbit anti-phospho-TBK-1/NAK (Ser172) (D52C2; Cell Signaling Technology, Danvers, MA, USA), rabbit anti-IKKi/IKKε (Abcam, Cambridge, UK), and rabbit anti-phospho-IKKε (Ser172) (D1B7; Cell Signaling Technology, Danvers, MA, USA) were used according to the manufacturers’ instructions. Rabbit polyclonal antibodies against RIG-I was as previously described [31]. For blocking the activity of type I interferons, a 1:50 dilution of Human Type I IFN Neutralizing Antibody Mixture (PBL Assay Science, Piscataway, NJ, USA) was used. In-house guinea pig anti-ZIKV-NS5 was prepared against ZIKV NS5 based on FB-GWUH-2016 sequence: A synthetic NS5 gene was ordered from GenArt (Thermo Fisher Scientific, Waltham, MA, USA), the NS5 coding sequence was cloned into a baculovirus expression vector GST-pBVboost to produce a chimeric GST-NS5 coding expression vector. Detailed gene expression, protein production, purification, and immunization protocols are described in Melén et al., 2017 [32]. 

### 2.3. Cells

Human embryonic kidney 293 (HEK293; ATCC, www.atcc.org) cells were maintained in Dulbecco’s modified Eagle’s medium (D-MEM) supplemented with HEPES (MP Biomedicals, Santa Ana, CA, USA), and human hepatocellular carcinoma HuH7 cells [33] were maintained in minimum Eagle’s medium-α (Invitrogen, Carlsbad, CA, USA). Cell media were supplemented with 10% heat-inactivated fetal bovine or calf serum (Biowest, Nuaillé, France or Integro, Zaandam, The Netherlands), penicillin/streptomycin and D-MEM also with L-glutamine. *Spodoptera frugiperda* (*Sf*9) cells, used for baculovirus expression, were maintained in TNM-FH medium (Sigma-Aldrich, St. Louis, MO, USA) as described previously [34].

### 2.4. Transfections, Cell Stimulations, and Infections

Plasmids were transfected into HEK293 cells with Lipofectamine2000 (Invitrogen, Carlsbad, CA, USA) or with TransIT-LT1 Reagent (Mirus Bio, Madison, WI, USA) and into Huh7 cells with TransIT-LT1 Reagent (Mirus Bio, Madison, WI, USA). To activate MxA promoter, IFN-α2b (IntronA; Merck Sharp & Dohme, Kenilworth, NJ, USA) IFNβ-1b (Bayer, Leverkusen, Germany), IFN-γ (Gibco), IFN-λ1 (Invitrogen), and TNF-α (Gibco, Thermo Fisher Scientific, Waltham, MA, USA ) were added in indicated concentrations into the cell culture media. For polyI:C stimulation, HEK293 cells were transfected overnight with TransIT-LT1 Reagent with indicated expression plasmids and pIFN-λ1-Luc reporter construct, followed by 7.3 µg/mL polyI:C (LMW, InvivoGen, San Diego, CA, USA) transfected with Lipofectamine2000. PolyI:C-induced cells were incubated overnight, and luciferase activities were measured.

### 2.5. Reporter Gene Assay

HEK293 cells were grown on 96-well plates and transfected at 70–80% confluency with promoter-luciferase constructs (20 ng/well), with RIG-I pathway expression plasmids (30 ng/well, except 3 ng/well for IKKε), and with ZIKV or HCV NS3/4A expression constructs (3–30 ng/well). RSV-Renilla (50 ng/well) was included as an internal control. Cells were harvested at indicated time points for Dual-Luciferase Reporter Assay System (Promega, Madison, WI, USA) or for twinlite Firefly and Renilla Luciferase Reporter Gene Assay System (Perkin Elmer, Waltham, MA, USA) according to the manufacturer’s instructions. The firefly luciferase results were normalized with the values of Renilla luciferase. All experiments were done in triplicates and repeated at least three times.

### 2.6. Immunoblotting

For immunoblotting, cells were transfected on 12-well plates with 400 ng per well expression plasmids for ∆RIG-I, IRF3, MAVS, TBK-1, and IRF3-5D, and 30 ng or 40 ng per well expression plasmid for IKKε. NS3, NS5, and HCV NS3/4A expression plasmids were transfected with 40 ng and 400 ng per well (or other indicated amounts). After overnight incubation, the cells were lysed on ice with Passive Lysis Buffer provided in the Dual Luciferase Assay Kit (Promega, Madison, WI, USA) or with a native lysis buffer as described previously [35]. Lysis buffers were supplemented with Complete Protease Inhibitor Cocktail (Roche, Basel, Switzerland) and PhosStop Phosphatase Inhibitor Cocktail (Roche, Basel, Switzerland). Proteins were separated on 4–12% or Any kD SDS-PAGE (BioRad, Hercules, CA, USA) and transferred onto an Amersham Protran 0.2 μm nitrocellulose blotting membranes (GE Healthcare Europe GmbH, Finland). Additionally, ZIKV E was also run under non-reducing conditions. Immunoblotting was done according to the manufacturer’s instructions for a particular antibody. Secondary antibodies were IRDye 800CW goat anti-rabbit IgG and IRDye 680RD goat anti-mouse IgG (LI-COR Biosciences, Lincoln, NE, USA). Membranes were scanned and analyzed with Odyssey Fc Imaging System (LI-COR Biosciences, Lincoln, NE, USA).

### 2.7. Immunofluorescence Microscopy

Huh7 cells were grown on glass coverslips and fixed with 3% paraformaldehyde in PBS at RT for 30 min. Cells were blocked and permeabilized with 0.5% BSA and 0.1% Triton X-100 in PBS at RT for 30 min. For localization studies in transfected cells, mouse anti-HA1.1 Epitope Tag (BioLegend, San Diego, CA, USA) was diluted 1:500 into 1% BSA in PBS and incubated at RT for 60 min. Secondary FITC-labeled anti-mouse antibodies (Jackson ImmunoResearch Laboratories, West Grove, PA, USA) were used according to the manufacturers’ instructions. After washing, coverslips were mounted on Mowiol (Sigma-Aldrich, St. Louis, MO, USA) and images were taken with a Leica TCS NT confocal microscope (Leica Microsystems, Wetzlar, Germany).

### 2.8. Production of GST Fusion Proteins in Sf9 Cells and GST-Pull-Down Assay

The production of GST and GST-tagged HCV NS2 and IAV NP in *Sf*9 cells was done using a baculovirus expression system that has been described previously [30]. The production of GST-tagged ZIKV NS5 in *Sf*9 cells was done according to the instructions by Airenne et al. [29]. For protein production, the cells were infected with GST and GST-tagged ZIKV NS5, HCV NS2, and IAV NP expressing baculoviruses for 48–72 h. Whole-cell extracts were prepared by lysing the cells in a lysis buffer (50 mM Tris-HCl pH 7.4, 150 mM NaCl, 5 mM EDTA, 1% Triton X-100) and protease inhibitors (Roche, Basel, Switzerland) on ice for 10 min and suctioning five times through a 25 G needle to disrupt DNA, followed by centrifugation (10,000× *g*, + 4 °C, 5 min). For pull-down experiments, 1 mg of soluble cellular protein samples were bound to 25 μL of Glutathione Sepharose 4B beads (GE Healthcare Europe GmbH, Finland) for 1 h at + 4 degrees and washed three times with the binding buffer (same as above but with 0.5% Triton X-100). The purity and quantity of each fraction were verified with Coomassie Blue staining on 12% SDS-PAGE (compared to known standard protein, bovine serum albumin). To produce radioactively labeled proteins for binding studies, in vitro-translated (TnT^®^ Quick Coupled Transcription/Translation Systems; Promega, Madison, WI, USA) MAVS, IKKε, and TBK1 were [^35^S]-labeled (Easy TagTM Express Protein Labeling Mix, PerkinElmer, Waltham, MA, USA) and allowed to bind to Sepharose-immobilized GST or GST-fusion proteins on ice for 60 min followed by washing three times with the binding buffer. GST and GST-fusion protein-bound, [^35^S]-labeled proteins were separated on 12% SDS-PAGE. The gels were fixed and treated with Amplify reagent (Amersham Biosciences, Little Chalfront, UK) as specified by the manufacturer followed by autoradiography.

For analysis of GST and GST-fusion protein-bound proteins by immunoblotting, proteins were separated on SDS-PAGE gels and transferred onto Immobilon-P membranes (polyvinylidene difluoride; Merck Millipore, Burlington, MA, USA) with an Isophor electrotransfer device (Hoefer Scientific Instruments, Holliston, MA, USA). The proteins were detected with rabbit anti-FLAG (F7425; Sigma-Aldrich, St. Louis, MO, USA), followed by secondary peroxidase-conjugated anti-rabbit IgG (DAKO). Detection was done on HyperMax films using an ECL Plus system (GE Healthcare Europe GmbH, Finland).

### 2.9. Statistical Analyses

For the luciferase assay data, the Student’s *t*-test in SPSS (IBM, Armonk, NY, USA ) was used to determine the statistically significant differences between the observed levels of inhibition compared with the control following the transfection of different amounts of ZIKV plasmids.

## 3. Results

### 3.1. Zika Virus Protein Expression in Mammalian Cells

For the expression of ZIKV structural proteins, a virus strain isolated in Brazil in 2015 was chosen. While these experiments were ongoing, a ZIKV isolate from an aborted fetus was grown and genetically characterized in 2016 [36]. Since this ZIKV isolate was evidently pathogenic in humans and it grew well in fetal brain, we decided to use this strain for the analysis of the functions of non-structural proteins. At the amino acid level, the structural proteins of these two isolates are 99.87% identical (two amino acid substitutions) and both represent the Asian lineage of Zika viruses.

**Figure 1:**
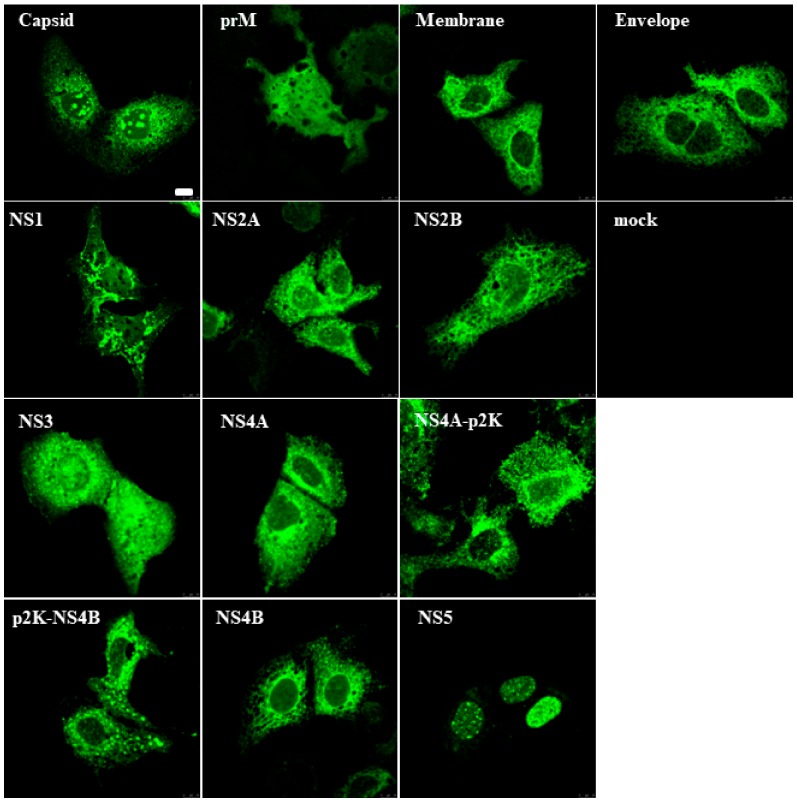
The Zika virus (ZIKV) protein expression in mammalian cells. Huh7 cells were transiently transfected with expression plasmids for various ZIKV proteins as indicated in the figure. After 24 h of transfection, the cells were fixed, stained with rabbit anti-HA antibody, and N-terminally HA-tagged ZIKV proteins were visualized with confocal laser microscopy. Bar, 10 μm.

Flavivirus proteins are expressed as a polyprotein that is processed by cellular and viral proteases into functional proteins and protein complexes. In this study, we focused to analyze the functions of individual ZIKV proteins on innate immune pathways. To verify the expression and intracellular localization of ZIKV proteins, individual ZIKV proteins were expressed in Huh7 hepatoma cells transfected with expression plasmids encoding N-terminally HA-tagged ZIKV proteins. Subcellular localization was monitored with confocal microscopy (Figure 1). All structural and non-structural ZIKV proteins were clearly detectable in transfected cells with an anti-HA antibody and showed distinct subcellular localizations. The majority of the expressed proteins were distributed in the cytoplasm, whereas ZIKV capsid protein formed distinct nuclear clusters and NS5 protein was localized mainly in the cell nucleus.

The flavivirus NS4A-2K-NS4B region contains a 2-kDa signal peptide designated p2K which spans the endoplasmic reticulum membrane. In DENV, the p2K directs the localization of NS4A and/or NS4B onto cellular membranes and it may be important for rearranging cytoplasmic membranes [37,38,39]. Since there is no information on the function of the ZIKV 2K peptide, both NS4A and NS4B were expressed with and without p2K. The presence of p2K sequence directed both proteins to more membranous structures in Huh7 cells (Figure 1). All proteins, except prM, NS2A, and NS2B, were detectable by immunoblotting in cell lysates from transfected HEK293 cells (Supplementary Materials Figure S1).

### 3.2. ZIKV NS5 Strongly Inhibits the Activation of IFN-λ1 and IFN-β Promoters

**Figure 2:**
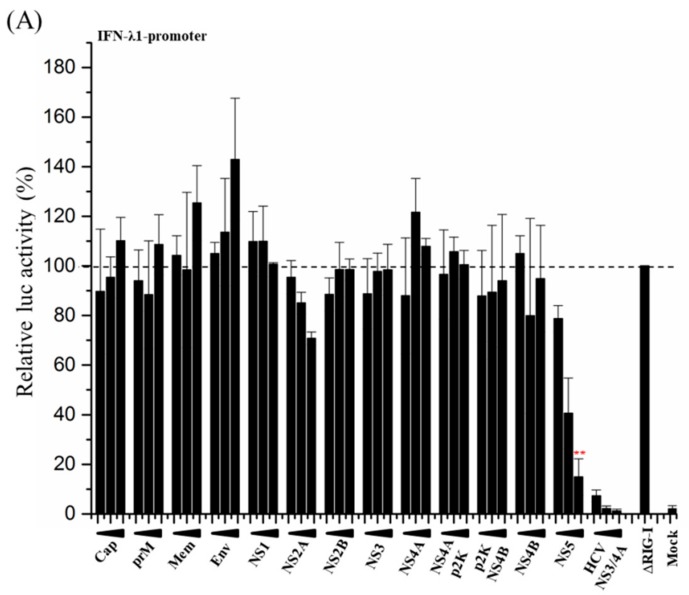

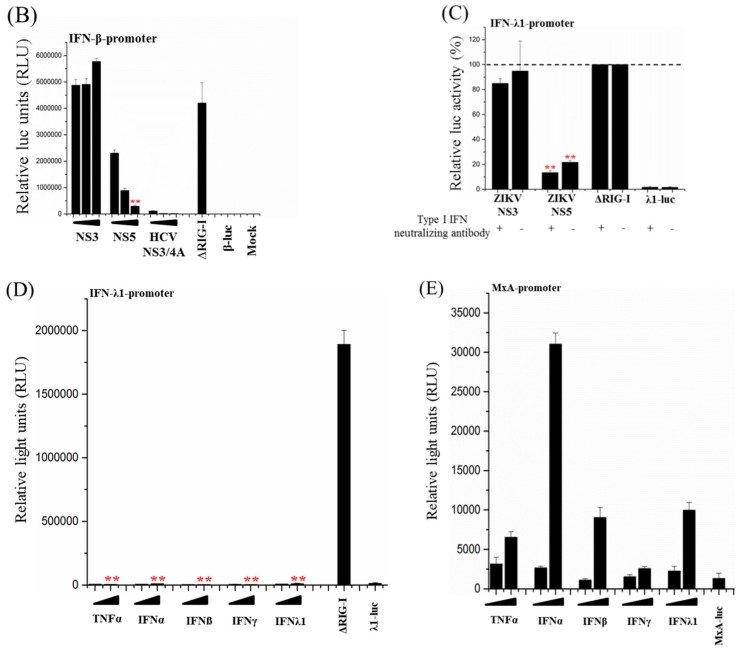
ZIKV NS5 protein inhibits RIG-I-induced interferon (IFN)-λ1 and IFN-β promoter activation. (**A**) Human embryonic kidney 293 (HEK293) cells were co-transfected with plasmids for ∆RIG-I, IFN-λ1-promoter-luciferase, and ZIKV expression plasmids, and luciferase (luc) activity was measured at 24 h after transfection. Luciferase activity induced by the positive control (∆RIG-I) was set to 100%. ** indicate a significant reduction in gene expression *p* < 0.05. (**B**) HEK293 cells were co-transfected with plasmids for ∆RIG-I, IFN-β-promoter-luciferase, ZIKV NS3 and NS5 expression plasmids, and the luc activity was measured at 24 h after transfection. An expression plasmid for HCV NS3/4A was used as a control for effective inhibition of IFN promoter activation. (**C**) HEK293 cells were co-transfected with plasmids for ∆RIG-I, IFN-λ1-promoter-luciferase, and ZIKV NS3 and NS5 expression plasmids, in the presence and absence of Human Type I IFN Neutralizing Antibody Mixture followed by measuring the luc activity at 24 h after transfection. (**D**) HEK293 cells were transfected with IFN-λ1 promoter luciferase reporter. Indicated cytokines were added 24 h post transfection (TNF-α: 10 ng/mL and 100 ng/mL, IFN-α2: 100 IU/mL and 1000 IU/mL, IFN-β: 100 IU/mL and 1000 IU/mL, IFN-γ: 100 IU/mL and 1000 IU/mL, IFN-λ1: 10 ng/mL and 100 ng/mL) and incubated for 24 h. The luc activities were measured and were compared with that of cells transfected with ΔRIG-I. (**E**) HEK293 cells were transfected with MxA promoter luciferase reporter. The same cytokines and respective amounts mentioned above were added at 24 h post transfection. ** indicate a significant reduction in promoter activation (*p* < 0.05). All experiments were repeated at least three times and a representative experiment is shown.

To study the effect of ZIKV proteins on the RIG-I pathway leading to the activation of type I and III IFN promoters, HEK293 cells were transfected with a plasmid encoding a constitutively active form of RIG-I (∆RIG-I), reporter plasmid constructs with the luciferase gene under IFN promoters, and expression plasmids for different ZIKV proteins. Overexpression of ∆RIG-I strongly induces the pathway, leading to the activation of the IFN-λ1 promoter (Figure 2A, set as 100%). A known strong inhibitor of this pathway, hepatitis C NS3/4A (HCV NS3/4A) which cleaves the MAVS protein and releases it from the mitochondrial membrane, was used as a control and it efficiently and dose-dependently inhibited ∆RIG-I-induced IFN-λ1 promoter activation. When co-transfected with increasing amounts of expression plasmids for ZIKV proteins, none of the structural proteins had any inhibitory effect on IFN-λ1 promoter activation. Of the non-structural proteins, NS2A inhibited up to 25% and NS5 showed remarkable dose-dependent, up to 80% inhibition of ∆RIG-I-induced IFN-λ1 promoter activation (Figure 2A). The same inhibitory effect of NS5 on IFN-λ1-promoter activation was detected when cells were stimulated with polyI:C (Supplementary Materials Figure S2).

To verify that the activation of type I IFN-β promoter is inhibited by our NS5 construct similarly as reported earlier [18], HEK293 cells were co-transfected with ∆RIG-I expression and IFN-β-promoter-luciferase reporter plasmids together with expression plasmids for ZIKV NS3, ZIKV NS5, and HCV NS3/4A. ZIKV NS3 had no inhibitory effect on ∆RIG-I-induced IFN-β promoter activation, whereas HCV NS3/4A efficiently inhibited the activation (Figure 2B). As with IFN-λ1 promoter activation, ZIKV NS5 showed strong dose-dependent inhibition of ∆RIG-I-induced IFN-β promoter activation (Figure 2B), confirming that the biological function of the isolate used in this study is similar to those reported previously.

**Figure 3:**
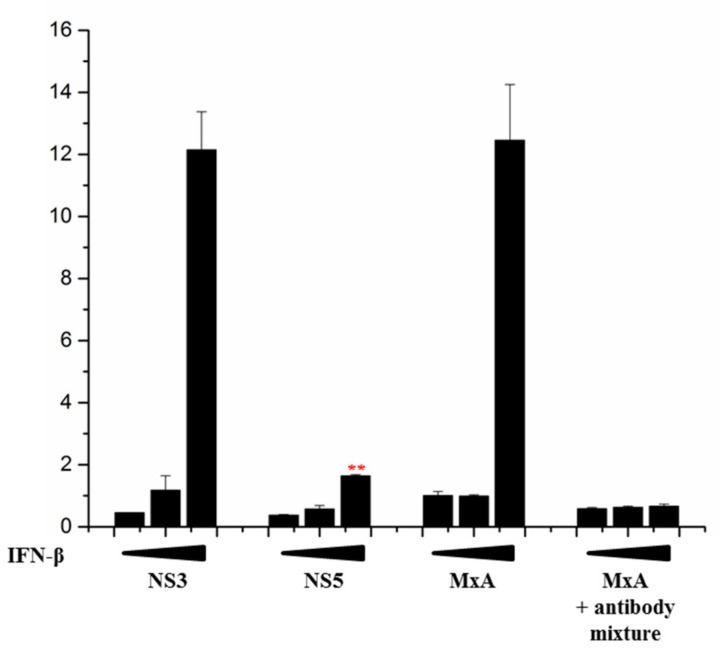
ZIKV NS5 inhibits IFN-induced pathway. HEK293 cells were co-transfected with plasmids for MxA-promoter-luciferase and ZIKV NS3 and NS5 expression plasmids. Six hours post-transfection, the cells were induced with IFN-β (0, 100, or 1000 IU/mL) and luc activity was measured at 24 h after transfection. Human Type I IFN Neutralizing Antibody Mixture that blocks the effect of IFN-β was included as a control. ** indicate a significant reduction in promoter activation (*p* < 0.05). All experiments were repeated at least three times and a representative experiment is shown.

Cells over-expressing ∆RIG-I secrete endogenous IFNs. These IFNs activate IFN-induced pathways as well as strengthen and regulate pathways leading to IFN-λ1-promoter activation, known as a positive feedback loop [40]. To verify that the observed inhibitory effect of ZIKV NS5 on the activation of IFN-λ1 and IFN-β promoters is acting directly on the RIG-I pathway and not on an IFN-induced feedback loop, the effect of putatively secreted endogenous IFNs was blocked with neutralizing antibodies. For this purpose, HEK293 cells were transfected with ∆RIG-I expression plasmid, IFN-λ1-promoter-luciferase reporter plasmid, and ZIKV expression plasmids for NS3 and NS5, and the transfected cells were treated with a mixture of neutralizing anti-human type I IFN antibodies that neutralizes the effect of IFN-α and IFN-β (Figure 2C). In the presence of type I interferon neutralizing antibodies, ZIKV NS5 showed a similarly strong 80% inhibition of IFN-λ1 promoter activation (Figure 2C), indicating that NS5 acts directly on the RIG-I pathway. The efficacy of the neutralizing antibody mixture is shown in Figure 3 where it strongly inhibited IFN-β-induced activation of IFN-inducible MxA-promoter.

To further rule out the activation of IFN-λ1 promoter by cytokines, the cells were transfected with the IFN-λ1-promoter-luciferase reporter construct and stimulated with cytokines TNF-α, IFN-α1, IFN-β, IFN-γ, and IFN-λ1 (Figure 2D). The MxA-promoter-luciferase construct was used as a control (Figure 2E). As shown in Figure 2D, none of the cytokines activated the IFN-λ1 promoter, whereas MxA-promoter was readily activated in response to stimulation with increasing amounts of cytokines. These results verify that the observed ZIKV NS5-mediated inhibition of the IFN-λ1 promoter is due to the interaction of ZIKV NS5 with the RIG-I pathway and not on the interferon-activated pathway(s).

### 3.3. ZIKV NS5 of Isolate FB-GWUH-2016 Inhibits MxA-Promoter Activation

ZIKV NS5 is known to inhibit IFN-induced antiviral response, e.g., MxA-activation, by inducing the degradation of STAT2 [17,18]. To confirm that the NS5 gene construct used in this study has a similar biological function, HEK293 cells were transfected with MxA-promoter-luciferase reporter plasmid and the promoter activation was induced by IFN-β. The MxA-promoter was strongly activated with increasing concentrations of IFN-β, and this activation was efficiently inhibited with a mixture of type I neutralizing antibodies (Figure 3). While ZIKV NS3 showed no inhibitory effect on IFN-β-induced MxA-promoter activation, ZIKV NS5 efficiently inhibited the IFN-β-induced activation of the MxA-promoter, verifying that the NS5 of the ZIKV isolate obtained from an aborted fetus has a similar inhibitory effect on IFN-induced responses as has been reported earlier for NS5 proteins of other ZIKV strains.

### 3.4. Full-Length ZIKV NS5 is Required for Its Inhibitory Activity on the RIG-I Pathway

**Figure 4:**
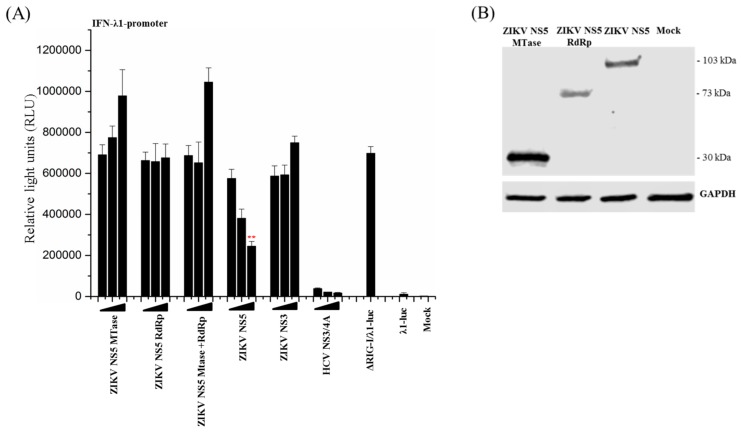
Full-length ZIKV NS5 is required for the inhibitory effect on IFN-λ1 promoter. (**A**) HEK293 cells were co-transfected with ΔRIG-I, IFN-λ1-promoter luciferase, and ZIKV MTase and RdRp, and full-length expression plasmids as indicated. The luc activity was measured at 24 h post transfection. ** indicate a significant reduction in promoter activation (*p* < 0.05). All experiments were repeated at least three times and a representative experiment is shown. (**B**) HEK293 cells were transfected with ZIKV NS5 MTase (30 kDa), RdRp (73 kDa), or full-length NS5 (103 kDa). The expression levels were detected by immunoblotting with anti-HA antibody.

The flavivirus NS5 proteins are composed of two functional domains: An N-terminal methyltransferase (MTase, aa 1–278) domain and a C-terminal RNA-dependent RNA polymerase domain (RdRp, aa 276–903) [41]. It has been reported that in the African lineage MR766 ZIKV NS5, which has been shown to inhibit IRF3 phosphorylation by blocking TBK1 activation, the full-length native NS5 protein is required for the inhibitory effect to occur [22]. To study whether the observed inhibitory effect of the Asian lineage FB-GWUH-2016 ZIKV NS5 on ΔRIG-I-induced IFN-promoter activation was associated with either one of the functional domains, the N-terminally HA-tagged domains were expressed separately or simultaneously, together with the IFN-λ1-promoter-luciferase reporter and ΔRIG-I. The full-length ZIKV NS5, ZIKV NS3, and the HCV NS3/4A were used as controls (Figure 4A). The expression of the domains was confirmed by immunoblotting (Figure 4B). Full-length NS5 and HCV NS3/4A strongly and dose-dependently inhibited the activation of IFN-λ1-promoter whereas the expression of separate MTase and RdRp domains alone or simultaneously displayed no inhibitory effect on the ΔRIG-I-induced IFN-λ1 promoter activation. The location of the tag was not relevant, since the results were the same with C-terminally myc-tagged MTase and RdRp expression constructs. This shows that the full-length, native NS5 protein is required for the inhibitory effect.

### 3.5. Activation of Both IRF3 and NF-ĸB Transcription Factors Are Inhibited by ZIKV NS5

**Figure 5:**
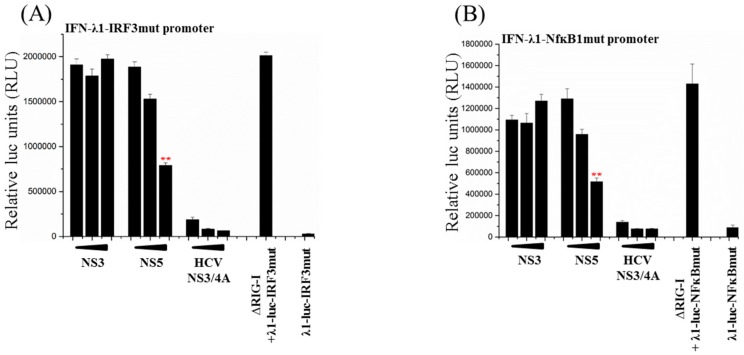
ZIKV NS5 inhibits IFN-λ1-promoter activation on both IRF3 and NF-ĸB pathways. HEK293 cells were co-transfected with expression plasmids for ∆RIG-I and ZIKV NS3 or NS5 plasmids, and with IFN-λ1-promoter-luciferase reporter plasmids which were mutated on (**A**) IRF3 or (**B**) NF-ĸB1 binding sites. An expression plasmid for HCV NS3/4A was used as a control. Luc activity was measured at 24 h after transfection. ** indicate a significant reduction in promoter activation (*p* < 0.05). All experiments were repeated at least three times and a representative experiment is shown.

The promoter area of the IFN-λ1 gene contains binding sites for IRFs (IRF3 and PRD1) and for NF-ĸB (two sites) [42,43]. To analyze whether either one or both of the RIG-I-pathway activated transcription factors, IRF3 or NF-ĸB, were inhibited by ZIKV NS5, HEK293 cells were co-transfected with the ∆RIG-I expression plasmid and IFN-λ1 promoter reporter constructs containing inactivating mutations on IRF3-binding or NF-ĸB binding sites (site 1, the most proximal NF-ĸB site) [43], and with expression plasmids for ZIKV NS3 and NS5 as well as HCV NS3/4A proteins. Overexpression of ∆RIG-I strongly activated both IRF3 (Figure 5A) and NF-ĸB-site (Figure 5B) -mutated IFN-λ1 promoters. HCV NS3/4A efficiently inhibited the activation of both of the mutated IFN-λ1 promoter-reporter constructs. ZIKV NS3 had no inhibitory effect on either one of the mutated IFN-λ1 promoter constructs, whereas ZIKV NS5 had a dose-dependent inhibitory effect on both IRF3-site and NF-ĸB-site-mutated IFN-λ1 promoters. These results indicate that ZIKV NS5 inhibits RIG-I-mediated activation of both IRF3 and NF-ĸB.

### 3.6. ZIKV NS5 Inhibits the RIG-I Pathway at Multiple Levels

**Figure 6:**
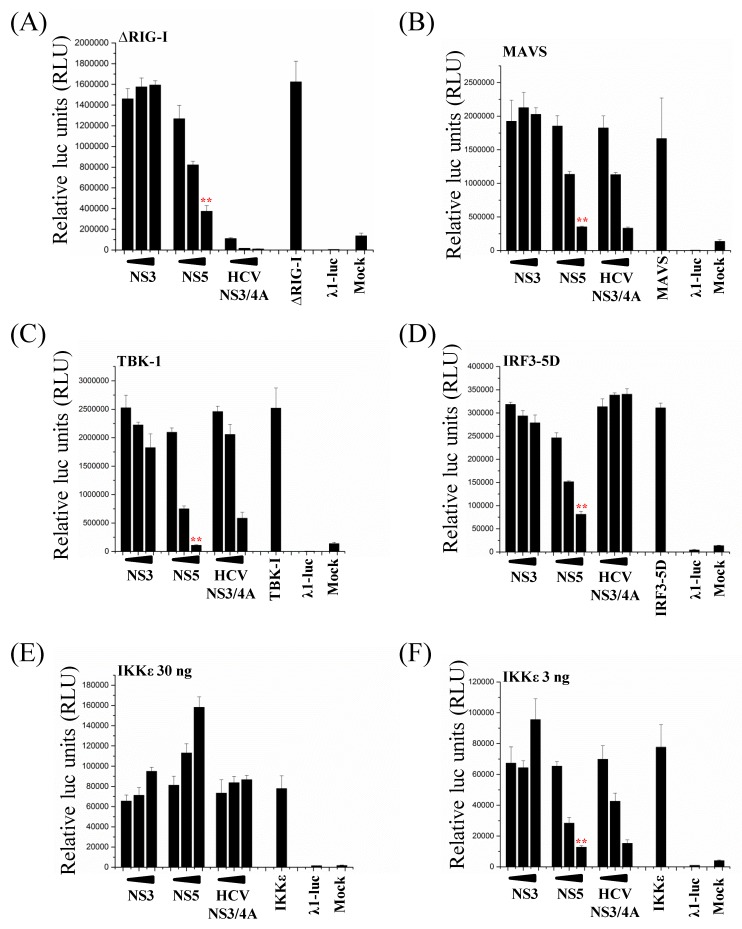
ZIKV NS5 inhibits IFN-λ1-promoter activation on all the components of the RIG-I pathway. HEK293 cells were co-transfected with expression plasmids for (**A**) ∆RIG-I, (**B**) MAVS, (**C**) TBK-1, (**D**) the constitutively active form of IRF3 (IRF3-5D), and (**E**, **F**) IKKε, and with expression plasmids for ZIKV NS3 and NS5. An expression plasmid for HCV NS3/4A was used as a control. Luc activity was measured at 24 h after transfection. ** indicate a significant reduction in promoter activation (*p* < 0.05). All experiments were repeated at least three times and a representative experiment is shown.

Overexpression of the main components of the RIG-I pathway leads to the activation of the IFN-λ1 promoter. To study at which level on the RIG-I pathway ZIKV NS5 exerts its inhibitory functions, HEK293 cells were co-transfected with ∆RIG-I, MAVS, TBK-1, IKKε, and a constitutively active form of IRF3 (IRF3-5D), together with ZIKV NS5 expression plasmid. ZIKV NS3 and HCV NS3/4A were used as putatively negative and positive controls, respectively. Overexpression of the pathway components led to a strong activation of the IFN-λ1 promoter (Figure 6). HCV NS3/4A protease complex, which degrades the MAVS protein [44], strongly inhibited ∆RIG-I, MAVS, TBK-1, IKKε, and, to a lesser extent, IRF3-5D-induced activation of IFN-λ1 promoter. ZIKV NS3 had no inhibitory effect on any of the pathway components. Surprisingly, ZIKV NS5 dose-dependently inhibited the activation of IFN-λ1 promoter by all the pathway components, indicating that the inhibitory effect of ZIKV NS5 may act at or downstream IRF3 activation. It is noteworthy that the inhibitory effect of ZIKV NS5 on IKKε overexpression-induced activation of the IFN-λ1 promoter was detected only with reduced amounts (from 30 ng per well to 3 ng per well) of IKKε expression plasmid, indicating that high-level expression of IKKε can override the inhibitory effect of NS5 (Figure 6, at the bottom).

### 3.7. NS5 Inhibits Phosphorylation of IRF3 and IKKε

ZIKV NS5 is known to inhibit STAT signaling by targeting STAT2 for degradation [17,18]. To determine whether ZIKV NS5 induces the degradation of any of the RIG-I pathway components or reduces the phosphorylation of TBK-1, IKKε, or IRF3, HEK293 cells were co-transfected with the expression plasmids for RIG-I pathway components together with ZIKV NS5 or NS3 expression plasmids. HCV NS3/4 was used as a control.

**Figure 7:**
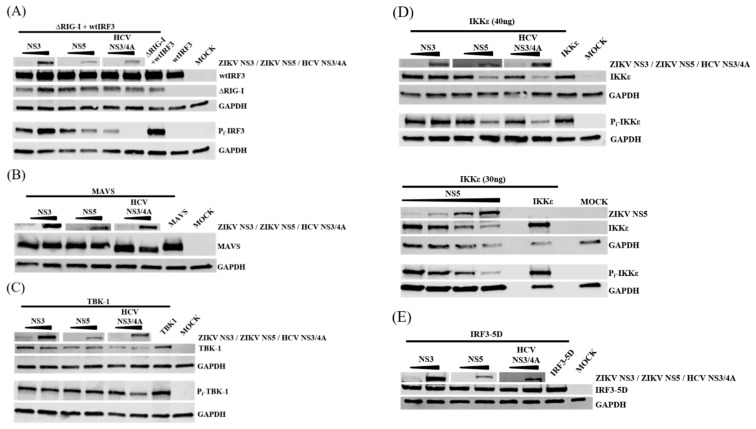
ZIKV NS5 inhibits phosphorylation of IRF3 and IKKε. HEK293 cells were co-transfected with expression plasmids for (**A**) ∆RIG-I (400 ng/well) and wtIRF3 (400 ng/well), (**B**) MAVS (400 ng/well), (**C**) TBK-1 (400 ng/well), (**D**) IKKε (40 ng/well and 30 ng/well) and (**E**) the constitutively active form of IRF3 (IRF3-5D) (400 ng/well), and with expression plasmids for ZIKV NS3 and NS5 (40 ng/well and 400 ng/well, for lower panel on IKKε NS5 plasmid amounts were 200 ng/400 ng/800 ng/1600 ng per well). An expression plasmid for HCV NS3/4A was used as a control. Expression levels and the phosphorylation status of the proteins were detected with immunoblotting.

To monitor IRF3 integrity and phosphorylation, cells were co-transfected with expression plasmids for ∆RIG and wtIRF3 (Figure 7A). As shown in the immunoblot analyses, ZIKV NS3, ZIKV NS5, and HCV NS3/4A had no effect on ∆RIG or wtIRF3 protein levels. However, ZIKV NS5 dose-dependently reduced IRF3 phosphorylation although to a lesser extent than HCV NS3/4A. These results indicate that NS5 exerts an inhibitory effect on the RIG-I pathway also upstream of IRF3 phosphorylation.

HCV NS3/4A degrades MAVS [44] and as shown in Figure 7B, MAVS was degraded to a slightly smaller protein form by HCV NS3/4A, whereas ZIKV NS3 or NS5 had no effect on protein amounts or cleavage of MAVS. Overexpression of TBK-1 resulted in autophosphorylation of itself. Co-expression of TBK-1 together with ZIKV NS3, ZIKV NS5, or HCV NS3/4A had no effect on protein levels or the extent of autophosphorylation of TBK-1 (Figure 7C). These results indicate that ZIKV NS5 is not targeting MAVS or TBK-1 for degradation.

Overexpression of IKKε also resulted in autophosphorylation of IKKε. As shown in Figure 7D (upper panel), co-expression of ZIKV NS3 had no effect on the expression or phosphorylation of IKKε, whereas both HCV NS3/4A and ZIKV NS5 reduced the expression levels and phosphorylation of IKKε. In further experiments this reduction was observed only with the amounts of the IKKε expression plasmid that showed inhibition of IFN-λ1-promoter activation in the luciferase assays; with higher amounts of IKKε expression plasmid, the NS5-related reduction in IKKε expression and phosphorylation were not that pronounced.

To further verify the effect of ZIKV NS5 on both the protein amount and the phosphorylation of IKKε, HEK293 cells were co-transfected with IKKε expression plasmid together with increasing amounts of ZIKV NS5 expression plasmid (Figure 7D, lower panel). Both the amount and the level of phosphorylation of IKKε were clearly reduced with increasing expression levels of ZIKV NS5. This indicates that ZIKV NS5 exerts an inhibitory function on IKKε by either directly or indirectly reducing the amount and phosphorylation of IKKε.

Luciferase assays indicated that ZIKV NS5 may also act downstream of IRF3 since ZIKV NS5 inhibited IRF3-5D-induced activation of the IFN-λ1 promoter. To monitor the effect of ZIKV NS5 on the protein level of IRF3-5D, HEK293 cells were co-transfected with expression plasmids for IRF3-5D and ZIKV NS3, ZIKV NS5, and HCV NS3/4A. As shown in Figure 7E, the protein levels of IRF3-5D remained the same regardless of co-expressed viral proteins.

Taken together, the results from luciferase and immunoblot assays indicate that, in addition to the inhibitory effect on the RIG-I pathway after IRF3 activation, ZIKV NS5 also exerts an inhibitory effect on ∆RIG-I-induced IRF3 phosphorylation, more specifically at the level of IKKε.

### 3.8. ZIKV NS5 Binds to IKKε

**Figure 8:**
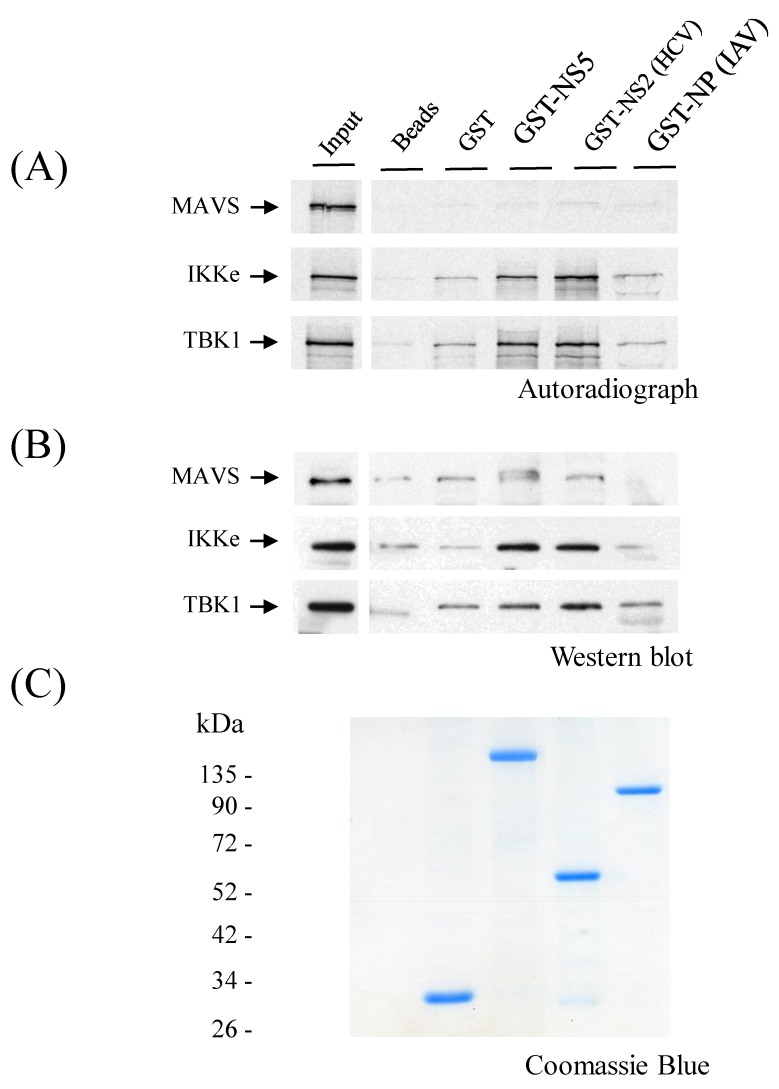
ZIKV NS5 binds IKKε. GST, GST-NS5 (ZIKV), GST-NS2 (HCV), and GST-NP (IAV) were produced in *Spodoptera frugiperda* (*Sf*9) cells using a baculovirus expression system. (**A**) [^35^S]methionine/cysteine-labeled, in vitro-translated MAVS, IKKε, and TBK-1 were allowed to bind to immobilized GST-NS5, GST-NS2, and GST-NP. Control lanes show in vitro-translated MAVS, IKKε, and TBK-1, and each lane represents one-tenth of the amount of the reticulate lysate that was used in each binding experiment. (**B**) HEK293 cells were transiently transfected in 6-well format with 4 μg expression plasmids for MAVS, IKKε, and TBK-1. At 24 h post-transfection, soluble cell extracts were prepared, and proteins in the cell extracts were allowed to bind to immobilized GST, GST-NS5, GST-NS2, and GST-NP. Bound proteins were separated on 12% SDS-PAGE, and the presence of MAVS, IKKε, and TBK-1 were analyzed by immunoblotting. (**C**) Coomassie Blue-stained gel is shown to visualize the amount of Glutathione Sepharose-bound GST and GST fusion proteins.

To find out whether ZIKV NS5 binds to the components of the RIG-I pathway, in vitro-translated or Huh7 cell-expressed MAVS, IKKε, and TBK-1 were allowed to interact with glutathione-Sepharose column-bound ZIKV GST-NS5. A known TBK1/IKKε interacting protein, HCV NS2 [30], and a non-binding protein, influenza A/Udorn/72 nucleoprotein (IAV NP), were used as positive and negative controls, respectively. As shown in Figure 8, GST or GST-fusion proteins bound in equal amounts into glutathione-Sepharose beads. MAVS did not bind to any of the three viral proteins, neither as an in vitro-translated protein nor as a cell-expressed protein (Figure 8A,B). In vitro-translated TBK-1 bound to GST-NS5 and GST-HCV-NS2, whereas the binding of cell-expressed TBK-1 showed similar (nonspecific) binding to GST-NS5 and control beads. Strikingly, a clear binding of IKKε to ZIKV NS5 and HCV NS2 was detected, both with in vitro-translated and Huh7 cell-expressed IKKε protein.

## 4. Discussion

ZIKV induces innate immune responses in infected cells since the expression of TLR3, RIG-I, and MDA5, as well as IFN-α, IFN-β, IFN-λ1, and MxA genes are enhanced [3,16,45]. Secreted IFNs protect cells from virus infection, and ZIKV infection is inhibited in cells pretreated with IFN-α, IFN-β, IFN-λ1, or IFN-γ [3,18,46]. Of these, IFN-λ1 is especially important in protecting epithelial barriers from virus infection [9] and, as shown earlier, ZIKV infection is prevented in placental trophoblasts which are secreting IFN-λ1 [45]. Thus, IFN-λ1 is important in protection from ZIKV infection since ZIKV spreads through epithelial barriers such as the skin, the blood-brain barrier, and the placenta. However, like other flaviviruses, ZIKV has the means to inhibit or delay the activation of innate immune responses. So far, most studies have focused on type I IFNs. In this study, we systematically analyzed the effect of all ZIKV proteins on the RIG-I pathway and type III IFN-λ1 promoter activation. We found that ZIKV NS5 protein efficiently inhibited the RIG-I pathway by interacting with IKKε followed by inhibition of phosphorylation of IRF3 and reduced activation of IFN promoters.

In previous studies, it has been shown that ZIKV NS5 inhibits polyI:C or RNA-induced activation of IFN-β [15,18,21] and NF-ĸB promoters [18]. Here, we also observed a strong inhibition of ∆RIG-I-induced activation of IFN-β promoter and of IFN-λ1 promoter and of polyI:C-induced activation of IFN-λ1 promoter by ZIKV NS5 protein. The inhibitory effect on the activation of IFN-λ1-promoter required a full-length NS5 protein, as has also been shown for TBK-1-related inhibition of IRF3 phosphorylation by the African lineage ZIKV NS5 [22]. We found that ZIKV NS5 inhibited ∆RIG-I-induced activation of both IRF3- and NF-ĸB transcription factors that coordinately regulate IFN-λ1 promoter activation (27). Consistent with a previous study on NS5-mediated inhibition of IFN-β promoter [21], we observed that ZIKV NS5 can also inhibit IFN-λ1 promoter activation induced by all components of the RIG-I pathway upstream or at the level of IRF3.

In addition to ZIKV NS5, we observed a weak inhibition of RIG-I-induced IFN-λ1 promoter activation by NS2A protein, whereas the other proteins were devoid of any inhibitory activity. Other researchers have, however, shown that ZIKV NS1, NS2A, NS2B, NS4A, and NS4B can inhibit poly(I:C)-induced activation of IFN-β promoter [18,20,21]. Our observations are well in line with the results of Hertzog and coworkers, who have shown that no other proteins apart from NS5 inhibit RNA-induced IFN-β promoter activation [15]. Some of the flavivirus NS proteins function together, like DENV NS2B-NS3 proteins which act as an active protease complex [47]. The functionality of different ZIKV protein combinations or complexes on innate immunity is presently not known, but, at least in our analyses, the p2K peptide did not provide any inhibitory effect on innate immunity for NS4A or NS4B proteins.

The exact molecular mechanisms of ZIKV proteins on the inhibition of IFN promoter activation are to some extent not known. Wu and coworkers have shown that ZIKV NS1 and NS4B inhibit IFN-β promoter activation by interacting with TBK-1 preventing its dimerization and phosphorylation [20]. Xia et al. [21] reported that IFN-β promoter activation was inhibited by NS2A, NS2B, and NS4B via blocking the phosphorylation of TBK-1. In addition, ZIKV NS4A suppressed IRF3 phosphorylation and NS5 was suggested to function on IRF3, possibly by interfering with the nuclear import of IRF3 [21]. Lin et al. reported that NS5 prevented IRF3 phosphorylation by direct binding to TBK-1 [22]. Our data on ZIKV NS5 inhibiting the activation of the IFN-λ1 promoter is well in line with the results described above, suggesting a function for ZIKV NS5 at or after activation of IRF3. However, in contrast to the results by Xia et al. [21], we observed that ZIKV NS5 inhibited the phosphorylation of IKKε and IRF3. In addition, we found a reduction in the amounts of IKKε with increasing expression levels of NS5, and furthermore, IKKε was also found to bind to NS5. In contrast to the data from Lin et al. [22] who demonstrated that NS5 can interact with TBK-1, we and Xia et al. [21] did not observe the inhibitory effect of NS5 on TBK-1. This discrepancy may be due to differences in the used ZIKV strains, cell types, or expression constructs, as well as differences in overall experimental conditions. Indeed, Esser-Nobis et al. [48] recently showed that infection with Zika viruses of different genetic lineages (Asian vs. African) led to differences in innate immune responses, resulting in differential IRF3 phosphorylation. In the above-mentioned studies, Wu et al. [20] and Lin et al. [22] used African ZIKV strain in their experiments, while Xia et al. [21] used both African and Asian lineage viruses, which both showed NS5-related inhibition of IFN-β-promoter activation.

Different flaviviruses are known to have different functions for the same/similar proteins. Flavivirus NS5 proteins have two active domains: A methyltransferase and an RNA-dependent RNA polymerase domain. Even though these biological functions are similar amongst flaviviruses, NS5 sequences of flaviviruses show up to 45% differences at amino acid level [17] allowing these proteins to have variable biological functions. For instance, DENV NS5 induces STAT2 degradation via E3 ubiquitin ligase [49], WNV NS5 inhibits IFNAR1 expression [50] and STAT1 phosphorylation [51], YFV NS5 binds to STAT2 and prevents its binding to ISRE sites [52], and ZIKV NS5 binds to STAT2 and induces its proteasomal degradation [17,18,19]. ZIKV NS5 also inhibits STAT1 phosphorylation which leads to reduced IFN-induced ISRE activation [15]. Even minor changes in amino acid sequences have been shown to impact these mechanisms. For example, a single amino acid substitution in ZIKV NS1 leads to a protein variant that targets the RIG-I pathway by inhibiting TBK-1 phosphorylation [21]. In addition, a single amino acid substitution in WNV NS2A protein leads to an attenuated virus phenotype with an inability to inhibit IFN-α/β production [53]. In our study, the NS genes originate from a highly pathogenic Asian lineage ZIKV strain isolated from fetal brain. Whether there was a selection towards a more fit/aggressive virus type with an exceptional ability to inhibit innate immune responses remains to be studied.

In our study, we found a direct interaction between ZIKV NS5 and IKKε. In addition, NS5 seemed to reduce the expression and phosphorylation of IKKε. Importantly, with higher expression levels of IKKε, the inhibitory effect of NS5 on IFN-λ1 promoter activation was abolished, suggesting that the stoichiometry of NS5 and IKKε has an impact on the inhibitory activity of NS5. Other viral proteins are also known to interfere with the functions of IKKε. For example, DENV NS2B/NS3 protein complex interacts with IKKε and blocks its kinase activity [54], MERS-CoV ORF4b protein inhibits the formation of MAVS and IKKε complexes [55], and arenavirus NP binds to and colocalizes with IKKε, preventing IKKε autophosphorylation and IKKε-induced IRF3 phosphorylation [56]. It remains to be studied whether the inhibitory effect of ZIKV NS5 on IFN gene expression is due to direct binding of NS5 with IKKε or whether NS5 targets some scaffold/modulating proteins on the IKKε signalosome.

ZIKV has a huge potential to efficiently spread among naïve human populations through Aedes species and possibly other mosquito vectors. This global threat and newly-discovered disease burden of ZIKV emphasizes the need to systematically study the role of individual ZIKV proteins in virus–host cell interactions. ZIKV NS5 is a crucial protein for the life cycle of the virus. Importantly, the ZIKV NS5 structure has already been resolved [41,57] facilitating the development of small molecular inhibitors that could be used as antiviral substances [58]. In this study, we describe a new phenomenon of how ZIKV NS5 protein interferes with the host innate immune system providing potentially novel targets for rational drug design.

## 5. Conclusions

The Zika virus is a zoonotic virus that usually causes a mild disease in humans. In 2013, Zika virus spread to the immunologically naïve population in the Americas and was found to cause severe neurological symptoms. In RNA virus infections, TLR and RLR signaling pathways are activated leading to the production of IFNs and activation of antiviral responses. Most pathogenic viruses, including the Zika virus, encode proteins that interfere with innate immune responses. Zika virus infects through epithelial barriers where IFN-λ1 is an important component of the host cell defense. Here, we show that the Zika virus NS5 protein is able to interfere with the activation of IFN-λ1 promoter, by targeting the IKKε kinase of the RIG-I pathway. A more detailed understanding of the functions of ZIKV proteins on host cells will likely enable us to design new antiviral substances against this newly emerged virus infection.

## Supplementary Materials

The following are available online at https://www.mdpi.com/1999-4915/11/11/1024/s1, Figure S1: Immunoblots from whole cell lysates from HEK293 cells, Figure S2: ZIKV NS5 inhibits polyI:C induced activation of IFN-λ1 promoter, Table S1: Primers used in this study.
