# Supplementary material for: Zika Virus Non-Structural Protein NS5 Inhibits the RIG-I Pathway and Interferon Lambda 1 Promoter Activation by Targeting IKK Epsilon
Source: Viruses. 2019 Nov 4;11(11):1024. doi: 10.3390/v11111024 (PMC6893776; doi:10.3390/v11111024)
Supplement: Supplementary file 1 [file viruses-11-01024-s001.zip › Supplementary Figure S1.pdf]

(A)

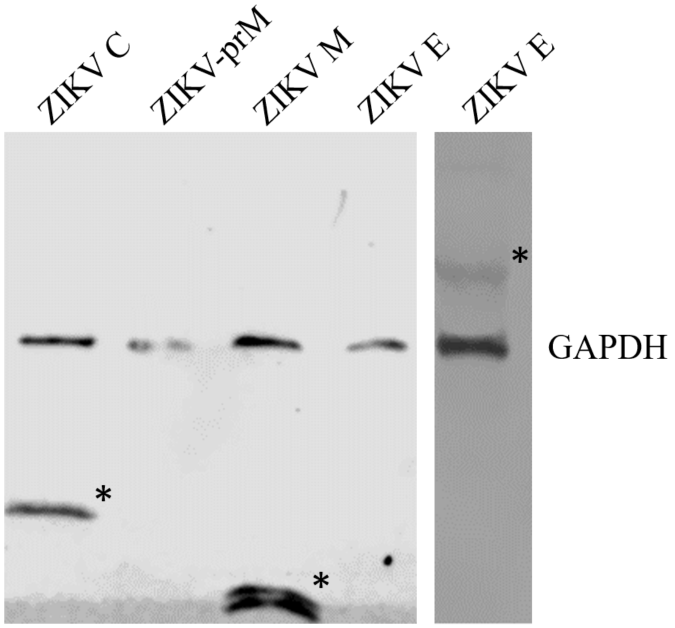

(B)

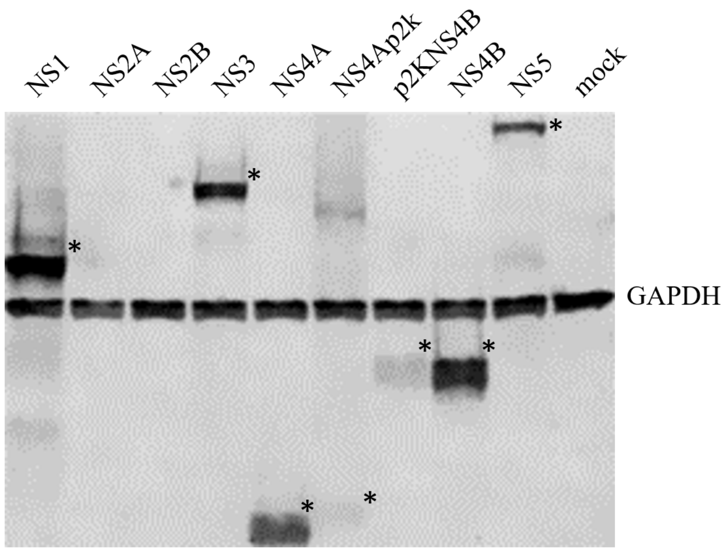

Figure S1. Immunoblots of whole cell lysates from HEK293 cells. HEK293 cells were transfected with 2400 ng of ZIKV expression plasmids on 12-well plates and incubated overnight. Expressed ZIKV proteins were detected with α-HA antibody, and GAPGH (37 kDa) was used as a loading control. (**A**) Two of the four structural HA-tagged proteins ZIKV-C (13.6 kDa), -prM (10.3 kDa), -M (8.48 kDa) and -E (54.5 kDa) were detected in immunoblot in reduced conditions (marked with an asterisk). ZIKV-E protein was detected under non-reducing conditions (right panel). (**B**) Seven of the nine non-structural proteins ZIKV-NS1 (40 kDa), -NS2A (24 kDa), -NS2B (13.8 kDa), -NS3 (68.5 kDa), -NS4A (13.7 kDa), -NS4Ap2K (16.1 kDa), -p2KNS4B (29.4 kDa), -NS4B (27.2 kDa), and -NS5 (102.9 kDa) were detected in immunoblot under reducing conditions (marked with an asterisk). Images are representative of at least three repetitions.
