# Supplementary material for: Zika Virus Non-Structural Protein NS5 Inhibits the RIG-I Pathway and Interferon Lambda 1 Promoter Activation by Targeting IKK Epsilon
Source: Viruses. 2019 Nov 4;11(11):1024. doi: 10.3390/v11111024 (PMC6893776; doi:10.3390/v11111024)
Supplement: Supplementary file 1 [file viruses-11-01024-s001.zip › Supplementary Figure S2.pdf]

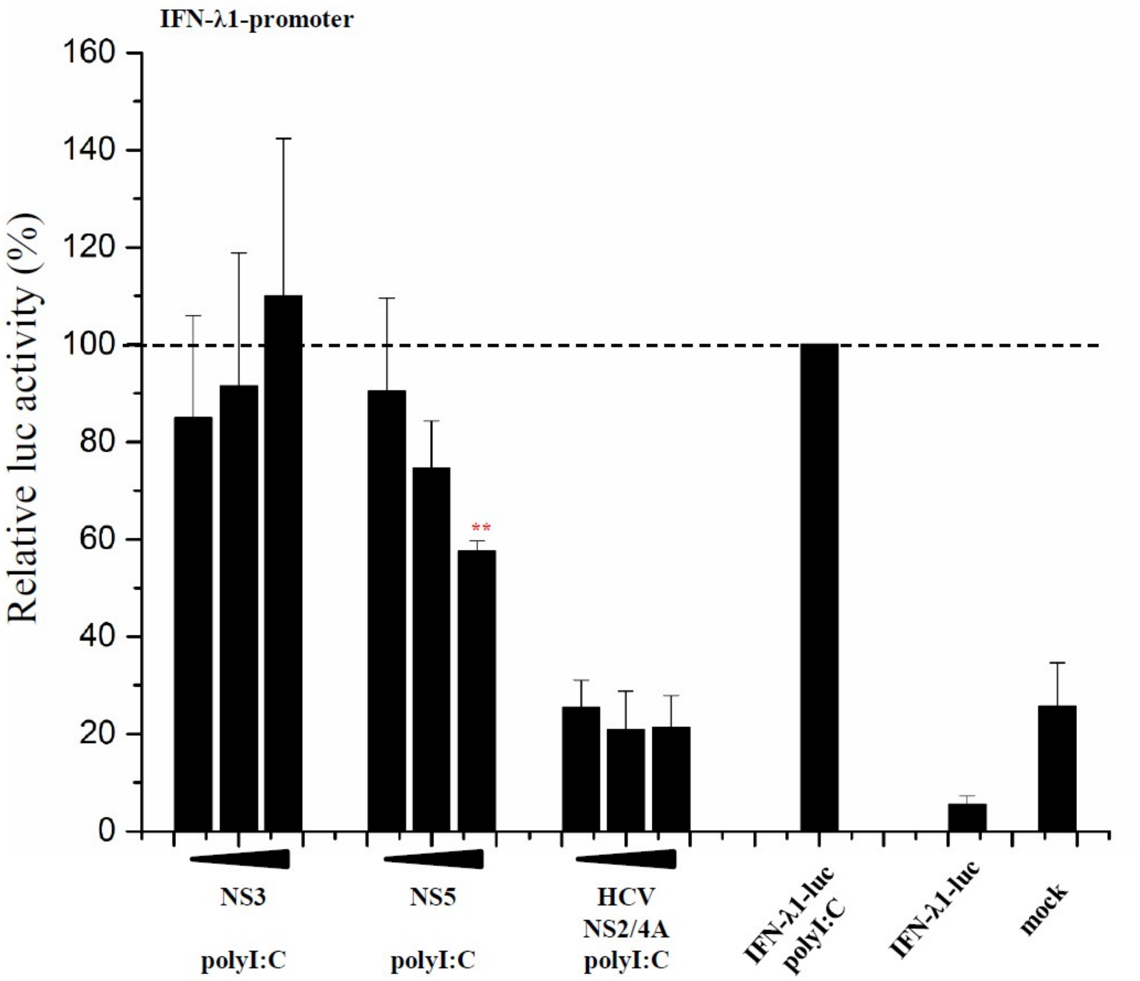

Figure S2. ZIKV NS5 inhibits polyI:C induced activation of IFN-λ1 promoter. PolyI:C is dsRNA that activates the RIG-I pathway. To verify the inhibitory effect of ZIKV NS5 on polyI:C-induced pathway, HEK293 cells were co-transfected with IFN-λ-promoter-luciferase reporter plasmids together with increasing amounts (3, 10 and 30 ng/well) of expression plasmids for ZIKV NS3, ZIKV NS5, and HCV NS3/4A proteins. After overnight incubation, the cells were stimulated with polyI:C for overnight and luciferase activities were measured. Results of two independent experiment were combined and luciferase activity induced by the positive control was set to 100%. ** indicate a significant reduction in promoter activation ($p < 0.05$).
