# Supplementary material for: Zika Virus Non-Structural Protein NS5 Inhibits the RIG-I Pathway and Interferon Lambda 1 Promoter Activation by Targeting IKK Epsilon
Source: Viruses. 2019 Nov 4;11(11):1024. doi: 10.3390/v11111024 (PMC6893776; doi:10.3390/v11111024)
Supplement: Supplementary file 1 [file viruses-11-01024-s001.zip › Table S1.pdf]

Table S1. Primers used in this study. Nucleotide numbering of Zika virus is based on the coding sequence of the isolate.

Gene		Primer (5′–3′)	Amplicon	Accession
CAP	Fwd	AAAGCG**GGATCC**AAAAACCCAAAAAAGAAATCC	1–375 (375)	KU365779.1
	Rev	AAAGCG**GGATCC**TTAGACCTCCGC		
prM	Fwd	AAAGCG**GGATCC**ACT'AGA'CGT'GGG	376–645 (270)	KU365779.1
	Rev	AAAGCG**GGATCC**TTATCTTCTAGATCTCCGTGCTTC		
MEM	Fwd	AAAGCG**GGATCC**GCTGTGACG	646–870 (225)	KU365779.1
	Rev	AAAGCG**GGATCC**TTAGCTGTATGCCGG		
ENV	Fwd	AAAGCG**GGATCC**ATCAGGTGC	871–2385 (1515)	KU365779.1
	Rev	AAAGCG**GGATCC**TTAATCAGCAGAGAC		
NS1	Fwd	AAAGCG**GGATCC**GATGTGGGGTGCTCG	2482–3537 (1056)	KU870645.1
	Rev	AAAGCG**GGATCC**TTATGCAGTCACCATTG		
NS2A	Fwd	AAAGCG**GGATCC**GGATCAACTGATCAC	3538–4215 (678)	KU870645.1
	Rev	AAAGCG**GGATCC**TTACCGCTTCCCACTCC		
NS2B	Fwd	AAAGCG**GGATCC**AGCTGGCCCCCTAG	4216–4605 (390)	KU870645.1
	Rev	AAAGCG**GGATCC**TTACCT'TTT'TCCAGTC		
NS3	Fwd	AAAGCG**AGATCT**AGTGGTGCTCTATGG	4606–6456 (1851)	KU870645.1
	Rev	AAAGCG**AGATCT**TTATCTCTTCCCAGCGGC		
NS4A	Fwd	AAAGCG**GGATCC**GGAGCGGCTTTTGG	6457–6840 (381)	KU870645.1
	Rev	AAAGCG**GGATCC**TTATCTTTGCTTTTCTG		
NS4Ap2K	Fwd	AAAGCG**GGATCC**GGAGCGGCTTTTGG	6457–6906 (450)	KU870645.1
	Rev	AAAGCG**GGATCC**TTAGGCGGTAATCAAGCC		
p2KNS4B	Fwd	AAAGCG**GGATCC**TCTCCCCAGGACAACC	6841–7659 (822)	KU870645.1
	Rev	AAAGCG**GGATCC**TTAACG'TCT'CTT'GAC'C		

NS4B	Fwd	AAAGCG**GGATCC**AATGAACTCGGATGG	6907–7659 (753)	KU870645.1
	Rev	AAAGCG**GGATCC**TTAACGTCTCTTGACC		
NS5	Fwd	AAAGCG**AGATCT**GGGGGTGGAACAGG	7660–10368 (2709)	KU870645.1
	Rev	AAAGCG**AGATCT**TTACAGCACTCCAGGTG		
NS5 MTase	Fwd	AAAGCG**AGATCT**GGGGGTGGAACAGG	7660-8494 (834)	KU870645.1
	Rev	AAAGCG**GGATCC**TTAAATGATCTTCATGTTGGG		
NS5 RdRp	Fwd	AAAGCG**GGATCC**ACCATGAAGATCATTGG	8485-10368 (1884)	KU870645.1
	Rev	AAAGCG**AGATCT**TTACAGCACTCCAGGTG		
GST	Fwd	ATAAGATCTACCATGTCCCCTATACTAGGTTATTGG		U13850.1
	Rev	TATAGATCTGGATCCGATCCACGCGGAACC		
